# The triglyceride-glucose index as an indicator of insulin resistance and cardiometabolic risk in Brazilian adolescents

**DOI:** 10.20945/2359-3997000000506

**Published:** 2023-01-17

**Authors:** Miriam Beatrís Reckziegel, Patrik Nepomuceno, Tania Machado, Jane Dagmar Pollo Renner, Hildegard Hedwig Pohl, Carlos Alberto Nogueira-de-Almeida, Elza Daniel de Mello

**Affiliations:** 1 Universidade Federal do Rio Grande do Sul Programa de Pós-graduação em Saúde da Criança e do Adolescente Porto Alegre RS Brasil Programa de Pós-graduação em Saúde da Criança e do Adolescente, Universidade Federal do Rio Grande do Sul (UFRGS), Porto Alegre, RS, Brasil; 2 Universidade de Santa Cruz do Sul Departamento de Ciências da Saúde Santa Cruz do Sul RS Brasil Departamento de Ciências da Saúde, Universidade de Santa Cruz do Sul (Unisc), Santa Cruz do Sul, RS, Brasil; 3 Universidade de Santa Cruz do Sul Programa de Pós-graduação em Promoção da Saúde Santa Cruz do Sul RS Brasil Programa de Pós-graduação em Promoção da Saúde, Universidade de Santa Cruz do Sul (Unisc), Santa Cruz do Sul, RS, Brasil; 4 University of Toronto Institute of Health Policy, Management and Evaluation Toronto ON Canada Institute of Health Policy, Management and Evaluation, University of Toronto (UofT), Toronto, ON, Canada; 5 University Health Network Lyndhurst Centre Kite Research Institute Toronto ON Canada Kite Research Institute, Lyndhurst Centre, University Health Network (UHN), Toronto, ON, Canada; 6 Universidade Federal de São Carlos Departamento de Medicina São Carlos SP Brasil Departamento de Medicina, Universidade Federal de São Carlos (UFSCar), São Carlos, SP, Brasil

**Keywords:** Insulin resistance, adolescent, diagnostic techniques, endocrine, endocrinology, metabolic syndrome

## Abstract

**Objective:**

To set cutoff points for the triglyceride and glucose index (TyG) as a marker of insulin resistance (IR) for the pediatric population.

**Subjects and methods:**

This was a cross-sectional study with schoolchildren population-based data using data of 377 schoolchildren age 10 to 17 years of both sexes. We studied metabolic variables associated with IR indicators, such as fasting insulin and blood glucose, to calculate the homeostatic model assessment (HOMA-IR), and we studied triglycerides (TG) to determine the TyG index. We obtained TyG cutoff values for IR using the receiver operation characteristic (ROC), with definitions of sensitivity (Sen), specificity (Spe), and area under the ROC curve (AUC), with the HOMA-IR as reference.

**Results:**

The cutoff points of the TyG index for IR in adolescents are 7.94 for both sexes, 7.91 for boys, and 7.94 for girls, indicating moderate discriminatory power. When we also considered anthropometric variables of excess weight [TyG-BMI (body mass index)] and visceral fat [TyG-WC (waist circumference)], these indexes reached AUC values higher than 0.72, enhancing their potential use for a good diagnosis.

**Conclusion:**

TyG has proven to be a useful instrument for identifying IR in adolescent health screening, with high discrimination capacity when added to anthropometric variables, making it a feasible and inexpensive option.

## INTRODUCTION

Insulin resistance (IR) presents as cells’ decreased sensitivity to insulin; in this condition, the body cells cannot correctly use the available insulin, leading to higher levels of blood glucose. IR is considered one of the main features of metabolic syndrome (MS), as it predisposes one to several disorders, such as elevated blood glucose, systemic arterial hypertension, and dyslipidemia ( 1 , 2 ). Early diagnosis of changes in the features of MS could facilitate preventive actions in public health ( 3 ). Offering an alternative method for diagnosis of IR, which is the pathophysiological basis for the development of MS, is one of this study’s objectives.

Among the methods for assessing IR, the hyperinsulinemic-euglycemic clamp, which analyzes the action of exogenous insulin, is considered the “gold standard”. However, it is difficult to carry out in clinical practice because of patient discomfort, high cost, and the technique’s difficulty and duration. Several surrogate indicators have been proposed, such as the IR Homeostatic Model Assessment (HOMA-IR), an indirect method, with the advantage of being calculated from one fasting blood sample for glucose and insulin ( 2 ). HOMA-IR, however, has some cutoff restrictions when used for children or adolescents; for instance, HOMA-IR varies significantly depending on age and pubertal stage, especially in adolescents, for both sexes, and there is no consensus on values for diagnosing IR in the pediatric population ( 4 ). Other indexes have emerged as a way to broaden the spectrum of techniques for analyzing IR in epidemiological studies, such as the triglyceride (TG)-to-high-density lipoprotein cholesterol (HDL-c) ratio, the fasting TG-to-glucose (G) ratio, and TyG ( 5 ).

Simental-Mendía and cols. ( 6 ) and Guerrero-Romero and cols. ( 7 ) proposed the TyG index, a logarithmic expression, as a low-cost marker for assessing IR ( 5 , 8 ). Studies have shown that increased TG can compromise muscle glucose metabolism, leading to decreased insulin sensitivity ( 9 , 10 ). However, values for age and sex have not been established and require further investigation, especially in the pediatric population ( 11 - 15 ).

We aimed to describe TyG as an indicator of IR in adolescents, defining cutoffs for the pediatric population based on HOMA-IR.

## SUBJECTS AND METHODS

This study population comprised female and male adolescents, age 10 to 17 years, enrolled in public and private schools in the urban and rural areas of Santa Cruz do Sul, Rio Grande do Sul, Brazil. From the subjects evaluated in the 2014/2015 period, we selected those who participated in a cohort with baseline in 2011/2012. We also used secondary data from the “Health of Schoolchildren – Phase III” survey, which assesses and monitors biochemical and hematological indicators and lifestyle-related risk factors every two years. The subjects came from 25 schools, stratified by conglomerate from more than 20,000 students; the sample was representative of the given municipality, respecting the proportionality of the region, zone, and administrative relation of the school as well as sex and age groups. The Human Research Ethics Committee of the University of Santa Cruz do Sul (UNISC) approved the study under protocol No. 1885957 (CAAE 63187316.0.0000.5343), and we obtained informed consent from all participants.

This study, linked to a cohort study, included 469 students who were participants of a cohort evaluated in 2014/2015; all participants were submitted to the same biochemical assessment protocols. As this is a predefined sample, we estimated the effect’s magnitude with statistical power of 80%, α = 0.05 and β = 0.2 (Spe = Sen*Spe/Sen = 0.262) ( 16 ). We calculated the sample size based on a 5% significance level and IR prevalence of 10.3% in Brazilian adolescents age 10 to 19 years ( 17 ).

Criteria for inclusion were completion of the data of the anthropometric and biochemical evaluations, signing of the assent form, and signing by their parents or guardians of the free and informed consent form allowing the use of the data in future studies. Exclusion criteria were inconsistent data, use of drugs that interfere with glucose and insulin metabolism, and insufficient blood sample for triplicate biochemical analysis.

To characterize the sample, we registered sex, age, ethnicity, socioeconomic level, and pubertal stage. A qualified professional of the same sex conducted the pubertal evaluation individually in a private environment, with the adolescents self-evaluating Marshall and Tanner’s ( 18 , 19 ) images, classifying them into maturational stages (I – prepubescent; II, III, and IV – pubescent; V – post-pubescent).

The techniques and instruments used in the collections were anthropometry, lipid profile, and IR markers. Regarding anthropometry, we measured weight, height, and waist circumference (WC) according to the World Health Organization’s (WHO’s) recommendations ( 20 ). Subsequently, we calculated the body mass index (BMI) and the nutritional status classified by the BMI Z-score according to the criteria the WHO proposed ( 20 ). We classified the subjects as underweight (Z-BMI > −1 SD), normal weight (≥ −1SD Z-BMI ≤ +1SD), overweight (BMI (z-score) > + 1 SD), and obese (Z-BMI > + 2 SD). We classified WC according to the criteria Fernández and cols. ( 21 ) established, with p ≤ 75 indicating normal risk and p > 75 indicating increased risk, according to sex and age. We also calculated waist/height ratio (WHtR) by dividing WC by height; we considered WHtR ≥ 0.5 a risk factor for abdominal obesity ( 20 ).

Blood was drawn from the brachial vein with the adolescent rested and having fasted for 12 hours, respecting biosecurity standards. We analyzed lipid profile [HDL-c, total cholesterol (TC), low-density lipoprotein cholesterol (LDL-c)], TG, and G on Miura One (I.S.E., Rome, Italy) using commercial DiaSys kits (DiaSys Diagnostic Systems, Germany). The cutoff points for defining the normality of lipid profile and blood glucose were those the Brazilian Society of Cardiology ( 22 ) and the International Diabetes Federation (IDF) ( 23 ) proposed: HDL-c ≥ 45 mg/dL, TC < 150 mg/dL, LDL-c < 100 mg/dL, TG < 100 mg/dL, and G < 100 mg/dL. We analyzed insulin in the serum sample by the chemiluminescence method on the ARCHITECT *i* 1000SR analyzer.

We determined the HOMA-IR according to the proposal of Matthews and cols. ( 24 ) [plasma G (mmol/dL) × plasma insulin (µUI/mL)/22.5] with a fixed cutoff of 3.16 ( 25 ), recommended by the I Guidelines of Prevention of Atherosclerosis in Children and Adolescents ( 26 ).

We calculated the IR index assessed by the ratio of TG to G, TyG, using the equation TyG = Log_n_ [TG (mg/dL) × fasting G (mg/dL)/2], and we expressed the results on a logarithmic scale ( 6 ). We also studied TyG adaptations using WC (TyG-WC) and BMI (TyG-BMI), suggested by Er and cols. ( 8 ), by multiplying TyG by BMI (TyG-BMI) and by WC (TyG-WC). For the TyG and other adapted indexes, we considered sex and age range (10 to 12 years, 13 to 14 years, and 15 to 17 years).

We conducted data analysis using the Statistical Package for the Social Sciences (SPSS), version 23.0 (IBM, Chicago, USA), and we checked all variables for normal distribution using the Shapiro-Wilk test. For sample characterization, we determined mean ± standard deviation, median, and interquartile range or amount (percentage). To compare the means between groups, we used Student’s *t* -test or the Mann-Whitney U test, and to compare the proportions according to age and sex, we used the chi-square test or Fischer’s exact test according to the data’s normality.

To estimate valid TyG cutoff points for the prediction of IR, we used the receiver operation characteristic (ROC), analyzing sensitivity (Sen) and specificity (Spe), considering the groups according to sex and age. We calculated the cutoff points as the maximum sum of Sen and Spe using the DeLong test and the Youden index in MediCalc 18.2.1 software. The area under the ROC curve (AUC) showed TyG cutoffs’ ability to distinguish adolescents with and without IR, predicted by the HOMA-IR cutoff, according to Borges’s classification ( 27 ).

## RESULTS

We evaluated 377 adolescents (55.7% girls) with a mean age of 12.79 ± 1.96 years. Regarding Tanner stages, 84.2% were between II and IV; 78.2% were Caucasian, and 54.2% were of socioeconomic class “C”. There were 132 adolescents with excess weight (63 boys); of those, 47 were obese (28 boys). Regarding excess abdominal fat assessed by WC, 23.1% were at increased risk (25.7% of boys and 21.0% of girls). Table 1 depicts the participants’ anthropometric and biochemical characteristics, according to sex. We noted significant differences between the sexes: boys had higher values of WC, WHtR, and G and lower LDL-c, TG, insulin, and HOMA-IR.

**Table 1:** Anthropometric and biochemical characteristics of the adolescents according to sex

Variables	Male (167)	Female (210)	p-value
Age	12.75 ± 2.01 (95% CI 12.45-13.06)	12.83 ± 1.91 (95% CI 12.57-13.09)	0.729
BMI-z	0.020 ± 1.025 (95% CI -0.137-0.176)	-0.156 ± 0.982 (95% CI -0.149-0.118)	0.734
WC-z	0.143 ± 1.087 (95% CI -0.023-0.309)	-0.114 ± 0.912 (95% CI -0.238-0.010)	0.013
WHtR-z	0.101 ± 1.056 (95% CI -0.060-0.262)	-0.802 ± 0.948 (95% CI -0.209-0.488)	0.081
HDL-c	63.21 ± 12.86 (95% CI 61.25-65.18)	60.92 ± 12.41 (95% CI 59.23-62.61)	0.071
LDL-c	79.93 ± 26.08 (95% CI 75.94-83.91)	85.63 ± 26.83 (95% CI 82.32-88.94)	0.023
TC	155.87 ± 33.79 (95% CI 150.71-161.03)	160.1 ± 33.86 (95% CI 155.49-164.7)	0.104
TG	65.46 ± 32.14 (95% CI 60.55-70.37)	75.66 ± 39.88 (95% CI 70.23-81.08)	0.030
G	92.56 ± 11.56 (95% CI 90.79-94.32)	89.36 ± 10.44 (95% CI 87.94-90.78)	0.005
Insulin	7.81 ± 4.46 9 (95% CI 7.13-8.49)	10.30 ± 5.74 (95% CI 9.51-11.08)	0.001
HOMA-IR	1.83 ± 1.3 (95% CI 1.63-2.03)	2.28 ± 1.3 (95% CI 2.1-2.45)	0.001

BMI-z: z score for body mass index; WC-z: z score for waist circumference; WHtR-z: z score for waist to height ratio; HDLc: high-density lipoprotein cholesterol; LDL-c: low-density lipoprotein cholesterol; TC: total cholesterol; TG: triglycerides; G: glucose, HOMA-IR: homeostatic model assessment of insulin resistance. Student’s t or Mann-Whitney test, expressed as mean ± SD and CI, confidence interval (CI).

Table 2 depicts TyG values and their sex and age distribution. Values differed between the sexes, and they progressively increased until 13 and 14 years of age and decreased from 15 to 17 years.

**Table 2:** Percentiles distribution of the triglycerides/glucose indexes (TyG) according to sex and age group

Characteristics		Percentiles												
		5P	10P	20P	25P	30P	40P	50P	60P	70P	75P	80P	90P	95P
General														
		7.17	7.39	7.62	7.68	7.74	7.83	7.97	8.07	8.20	8.26	8.34	8.53	8.80
Sex														
	M	7.06	7.33	7.53	7.61	7.67	7.79	7.95	8.04	8.17	8.24	8.29	8.49	8.82
	F	7.29	7.51	7.68	7.74	7.79	7.90	8.00	8.09	8.22	8.30	8.36	8.54	8.77
Age group														
	10 to 12 years	7.11	7.35	7.56	7.66	7.72	7.82	7.95	8.03	8.14	8.18	8.25	8.46	8.67
	13 to 14 years	7.34	7.51	7.68	7.71	7.79	7.92	8.00	8.13	8.27	8.32	8.39	8.54	8.81
	15 to 17 years	7.12	7.37	7.62	7.65	7.74	7.83	8.00	8.15	8.26	8.35	8.48	8.85	9.13

M: male; F: female. TyG percentiles defined by the ROC curve, HOMA-IR (3.16) used as reference.

The AUC was 0.64 for the group as a whole; the positive predictive value was 13.79%, the negative predictive value was 86.21%, and the Youden index was 0.2546. AUC was 0.75 and 0.59 for boys and girls, respectively. Table 3 presents the TyG cutoff points and corresponding Sen, Spe, and AUC for IR for the total sample as well as divided by sex and age group. The TyG cutoff values are ≥7.94 (Sen 75.0%, Spe 50.5%) for all participants, ≥7.91 (Sen 92.9%, Spe 51.0%) for boys, and ≥7.94 (Sen 71.1%, Spe 48.3%) for girls. We observed an increase in the age group 10 to 12 years compared to 13 to 14 years (≥8.07 x ≥8.48) and a decrease in the age group 15 to 17 years (≥7.93).

**Table 3:** Values for cutoff points for the triglycerides/glucose index (TyG) for insulin resistance, with sensitivity and specificity, according to sex and age group

	TyG cutoff	Sensitivity (95% CI)	Specificity (95% CI)	AUC (95% CI)	Youden
Total	≥7.94	75.0 (61.1-86.0)	50.5 (44.9-56.0)	0.64 (0.59-0.69) **	0.2546
Male	≥7.91	92.9 (66.1-99.8)	51.0 (42.8-59.1)	0.75 (0.67-0.81) **	0.4384
Female	≥7.94	71.1 (54.1-84.6)	48.3 (40.6-56.0)	0.59 (0.52-0.66)	0.1931
10-12 years	≥8.07	60.9 (38.5-80.3)	67.5 (59.7-74.7)	0.64 (0.57-0.71) **	0.2837
13-14 years	≥8.48	35.3 (14.2-61.7)	91.3 (83.6-96.2)	0.63 (0.53-0.72)	0.2660
15-17 years	≥7.93	83.3 (51.6-97.9)	49.3 (37.4-61.3)	0.65 (0.54-0.75) *	0.3265

TyG: triglyceride/glucose index; CI: confidence interval; AUC: area under the curve.

DeLong et al. test (1988), Youden index-defined cutoff points.

* p < 0.05.

** p < 0.001.

From the definition of TyG cutoff points for IR, Table 4 presents a comparison of adolescents with IR to those with no insulin resistance (NIR). The cutoff points established for IR-differentiated anthropometric and biochemical variables between the groups, IR and NIR, when we analyzed the group as a whole and the male subjects. For the girls, this difference is only significant in the biochemical variables. Adolescents classified with IR had excess weight (83 IR *versus* 49 NIR) and localized fat (assessed by WC – 53 IR *versus* 34 NIR) as well as an unhealthy biochemical profile (inadequate for TC in 137 adolescents with IR; LDL-c in 56; TG in 54; G in 43).

**Table 4:** Variables analyzed according to the cutoff points for insulin resistance and the triglyceride/glucose index (TyG), according to sex

TyG	Male (≥7.91)		Female (≥7.94)		Total	
	NIR	IR	NIR	IR	NIR	IR
	79	88	94	116	173	204
BMI-z	-0.196 ± 0.946	0.213 ± 1.059 **	-0.100 ± 0.864	0.524 ± 1.067	-0.144 ± 0.901	0.122 ± 1.064 **
WC-z	-0.057 ± 1.130	0.322 ± 1.020 *	-0.241 ± 0.805	-0.010 ± 0.981	-0.157 ± 0.969	0.133 ± 1.009 **
WHtR-z	-0.567± 1.035	0.242 ± 1.060	-0.209 ± 0.850	0.024 ± 1.013	-0.139 ± 0.939	0.118 ± 1.036 *
HDLc	65.90 ± 13.61	60.81 ± 11.72 **	61.69 ± 13.44	60.31 ± 11.54	63.61 ± 13.65	60.53 ± 11.59 *
LDLc	75.05 ± 22.86	84.31 ± 28.08 *	82.32 ± 28.00	87.43 ± 24.88	79.00 ± 25.96	86.09 ± 26.29 **
TC	148.39 ± 32.12	162.59 ± 34.03 *	153.60 ± 34.13	166.72 ± 29.30 **	151.22 ± 33.23	164.94 ± 31.41 ***
TG	42.61 ± 9.88	87.30 ± 33.15 ***	50.21 ± 12.77	95.29 ± 41.62 ***	46.74 ± 12.12	91.84 ± 38.31 ***
Glucose	88.62 ± 10.88	96.10 ± 11.06 ***	85.45 ± 11.24	92.54 ± 8.57 ***	86.90 ± 11.15	94.08 ± 9.86 ***
Insulin	6.20 ± 3.00	9.26 ± 5.05 ***	9.14 ± 4.93	11.25 ± 6.19 **	7.80 ± 4.40	10.39 ± 5.80 ***
HOMA-IR	1.36 ± 0.70	2.25 ± 1.56 ***	1.92 ± 1.05	2.57 ± 1.43 ***	1.66 ± 0.95	2.43 ± 1.49 ***

BMI-z: z score for body mass index; WC-z: z score for waist circumference; WHtR-z: z score for the waist-height rate; HDLc: high-density lipoprotein cholesterol; LDLc: low-density lipoprotein cholesterol; TC: total cholesterol; TG: triglycerides; HOMA-IR: homeostatic model assessment of insulin resistance; TyG: triglyceride/glucose index; NIR: no insulin resistance. Student’s t or Mann-Whitney tests, expressed as mean ± SD and CI, confidence interval (CI).

* < 0.05.

** < 0.01.

*** p < 0.0001.

To improve the diagnostic curves of metabolic risk-related IR, we added excess weight and visceral obesity variables (BMI and WC) as well as age and sex to TyG for the preliminary analyses, as recommended for the adult population ( 8 , 28 , 29 ). We identified greater predictive power in all categories studied after analyzing TyG jointly with WC and with BMI ( Table 5 ).

**Table 5:** Comparison of the triglycerides/glucose (TyG), triglycerides/glucose and waist circumference (TyG-WC), and triglycerides/glucose and body mass indexes (TyG-BMI) to predict insulin resistance according to sex and age group

	General	Male	Female	10 to 12 years	13 to 14 years	15 to 17 years
**TYG**						
Cutoff point	≥ **7.94**	≥ **7.91**	≥ **7.94**	≥ **8.07**	≥ **8.48**	≥ **7.93**
Sen	75.0 (61.1-86.0)	92.9 (66.1-99.8)	71.1 (54.1-84.6)	60.9 (38.5-80.3)	35.3 (14.2-61.7)	83.3 (51.6-97.9)
Spe	50.5 (44.9-56.0)	51.0 (42.8-59.1)	48.3 (40.6-56.0)	67.5 (59.7-74.7)	91.3 (83.6-96.2)	49.3 (37.4-61.3)
AUC (95%CI)	**0.64 (0.59-0.69)** **	**0.75 (0.67-0.81)** **	**0.59 (052-0.66)**	**0.64 (0.57-0.71)** *	**0.63 (0.53-0.72)**	**0.65 (0.54-0.75)** *
**TYG-WC**						
Cutoff point	**<555.00**	**<577.08**	**<551.89**	**<526.64**	**<615.82**	**<624.70**
Sen	80.8 (67.5-90.4)	85.7 (57.2-98.2)	81.6 (65.7-92.3)	91.3 (72.0-98.9)	58.8 (32.9-81.6)	75.0 (42.8-94.5)
Spe	64.3 (58.8-69.5)	69.3 (61.3-76.5)	68.0 (60.5-74.9)	63.1 (55.1-70.6)	85.9 (77.0-93.3)	85.6 (73.0-91.2)
AUC (95%CI)	**0.78 (0.74-0.82)** **	**0.81 (0.74-0.86)** **	**0.79 (0.73-0.85)** **	**0.80 (0.73-0.85)** **	**0.74 (0.65-0.82)** *	**0.82 (0.73-0.90)** **
**TYG-BMI**						
Cutoff point	**<168.95**	**<177.84**	**<168.64**	**<168.01**	**<181.15**	**<197.85**
Sen	80.8 (67.5-90.4)	85.7 (57.2-98.2)	79.0 (68.2-90.4)	87.0 (66.4-97.2)	70.6 (44.0-89.7)	66.7 (34.9-90.1)
Spe	65.9 (60.4-71.0)	75.2 (67.5-81.8)	65.7 (58.1-72.8)	73.1 (65.6-79.8)	79.4 (69.6-87.1)	84.9 (74.6-92.2)
AUC (95%CI)	**0.79 (0.74-0.83)** **	**0.84 (0.78-0.90)** **	**0.77 (0.70-0.82)** **	**0.83 (0.76-0.88)** **	**0.74 (0.65-0.82)** *	**0.76 (0.66-0.85)** *

TYG: triglycerides/glucose index; Sen: sensitivity; Spe: specificity; TYG-WC: triglycerides/glucose and waist circumference; TyG-BMI: triglycerides/glucose and body mass index; AUC: area under the curve. DeLong et al. (1988) test Youden index-defined cutoff points.

* p < 0.05.

** p < 0.001.

## DISCUSSION

The prevalence of MS and IR in the pediatric population is increasing worldwide ( 30 , 31 ). Studies with adult populations from various countries have associated TyG with IR, MS, and cardiovascular risk. Lee and cols. ( 32 ) and Irace and cols. ( 33 ) found a stronger correlation of TyG than of HOMA-IR with arterial stiffness. Compared to hyperinsulinemic-euglycemic clamp, TyG was more accurate ( 34 - 37 ).

Several studies have shown TyG’s clinical advantages for the diagnosis of IR in adults ( 2 , 33 , 36 ). However, in few studies, researchers have examined cutoff values of the indexes in the pediatric population ( 10 , 12 - 14 ). We found only one such study, conducted in Brazil ( 38 ).

We therefore intended to determine the distribution by TyG percentile as an indirect index of IR, its cutoff values for Brazilian adolescents, aiming to screen risk groups for IR and, as a consequence, for MS. The analysis of the ROC curves of the TyG index for IR, according to the HOMA-IR index, to find cutoff points valid for this population was also an objective.

Considering the variability between HOMA-IR cutoffs (2.0 to 3.43) suggested by multiple studies ( 39 - 41 ), we chose to use the fixed point 3.16 ( 25 ), which is widely used in scientific publications and recommended by the I Guidelines for the Prevention of Atherosclerosis in Children and Adolescents ( 26 ). The graphic illustration of the AUC made it possible to divide the population into healthy and unhealthy, indicating a diagnostic test’s discriminative power, with 1.0 being the maximum value and values below 0.50 indicating non-discrimination ( 27 ). TyG AUC for IR in the adolescents evaluated was 0.64 (0.59-0.69), demonstrating sufficient discriminative power. However, when added to anthropometric variables, these indexes reached values higher than 0.79, with good discrimination power, increasing the potential usefullness for the diagnosis of MS.

The TyG cutoff found in this study was ≥ 7.94 (AUC = 0.64), diagnosing IR in 54.3% of the subjects. This result was somewhat similar to that of the study by Vieira-Ribeiro and cols. ( 38 ), who identified TyG ≥ 7.88 (AUC = 0.63) in Brazilian children age 4 to 7 years, finding 42.3% of IR. Gesteiro and cols. ( 42 ), in a study with newborns from Spain, stated that TyG had good discriminatory power to diagnose IR. Kang and cols. ( 13 ), studying Korean adolescents, comparing TyG to other IR markers, found TyG ≥ 8.18.

Rodríguez-Morán and cols. ( 14 ), Guerrero-Romero and cols. ( 43 ), and Simental-Mendía and cols. ( 44 ) found lower values than we did, which can be attributed to the way the formula is used and when the calculation is carried out using TyG with a log function instead of Ln (natural log) ( 6 ). However, regardless of the TyG calculation method, there is variability between the cutoff points suggested by various authors in studies with children and adolescents, ranging from ≥ 7.80 ( 42 ) to ≥ 8.66 ( 15 ) and of TyG ≥ 4.55 ( 43 ) to ≥ 4.75 ( 14 ). These variations may be related to various characteristics of the population studied and the study conducted, such as age group, maturation stage, mixed sex versus female or male, different ethnic groups, obesity versus healthy weight, sample size, and different reference standards.

Considering sex and age group, Angoorani and cols. ( 10 ), in a study with characteristics similar to ours, evaluated TyG as one of the predictors of MS and obtained cutoff points of TyG ≥ 8.33 overall; ≥ 8.33 and ≥ 8.47 for boys and girls, respectively; ≥ 8.47 for ages 7 to 12 years; and ≥ 8.34 for 13 to 18 years. In this study, we found lower cutoff points: TyG ≥ 7.94 in general; ≥ 7.94 and ≥ 7.91 for girls and boys, respectively; ≥ 8.07 for ages 10 to 12 years; ≥ 8.48 for ages 13 to 14 years; and ≥ 7.93 for ages 15 to 17 years. However, TyG rates increase in early adolescence and subsequently decrease. Lee and cols. ( 45 ) stressed the importance of recognizing physiological and non-physiological changes in IR in this age group because insulin sensitivity significantly decreases with puberty, as fasting insulin increases approximately by 50% ( 40 ), causing a natural state of “physiological IR,” regardless of changes in body composition ( 14 ). We therefore noted a gradual increase in IR until the age of 12-13 years, which reaches a plateau, with subsequent reduction to pre-pubertal values ( 46 ) in girls and boys.

To characterize better the changes found in IR in the pediatric population, Mohd-Nor and cols. ( 12 ) considered the pubertal stage and ethnic groups, defining TyG ≥ 8.52 for screening and diagnosis of IR in adolescents classified as Tanner stages II and IV. In our study, 84.2% of adolescents met that criterion, and the reduction of IR after 15 years of age was evident, similar to the study by García Cuartero and cols. ( 47 ), in which most were already Stage V. These changes seen in puberty can be explained by the 30% clearance of glucose that occurs from Stages II to IV, peaking in Stage III and them returning to pre pubertal levels in Stage V ( 41 ).

Abdominal fat resulting from sexual maturation, early menarche, and reduced testosterone in boys with obesity may be associated with higher IR ( 48 ). According to Arslanian and cols. ( 49 ), obesity might have a greater effect on insulin sensitivity in youth than in phenotypically similar adults ( *i.e.* , in terms of sex, race, BMI, and body adiposity), especially regarding visceral fat deposition ( 14 ). Puberty affects fat oxidation, which would explain changes in IR ( 50 ) because at this stage, physiological redistribution of fat from the extremities to the trunk occurs, especially in girls ( 17 , 51 ). In this study, 35.0% of adolescents had excess weight (13.0% of them with obesity), lower than the rates reported in other studies with children and adolescents, which may explain the lower cutoffs found.

Considering obesity’s determining role for IR and to improve TyG Sen and Spe indexes, Zheng and cols. ( 28 ) suggested adding variables of excess weight or visceral fat to this index. Er and cols. ( 8 ) and Hameed ( 29 ) therefore evaluated the potential use of TyG and its related indexes (TyG-WC and TyG-BMI) in adults and correlated with HOMA-IR, presenting TyG-BMI as a better indicator for IR, corroborated by Almeda-Valdés and cols. ( 52 ) in a study with a hyperinsulinemic-euglycemic clamp, with TyG-BMI as the index of higher Sen and Spe. Similar to that found in this study, the first to use these related indexes in adolescents, the power of IR discrimination increased in all categories analyzed, generating TyG ROC curves with 0.64 AUC overall, 0.59 for girls, and 0.75 for boys, for TyG-WC of 0.78, 0.79, and 0.81 and TyG-BMI of 0.79, 0.77, and 0.84, respectively.

Therefore, TyG-WC and TyG-BMI showed better performance in IR recognition and can be considered clinically useful substitutes in this diagnosis because they combine GT, fasting G, and adiposity, parameters well validated in IR recognition ( 8 ). In our investigation, TyG-BMI performed more efficiently in some categories than TyG-WC in identifying IR; however, both had good discriminating power. BMI is simple to measure and is commonly adopted as a useful indicator of general obesity and other metabolic abnormalities although it does not distinguish body fat from fat-free mass whereas abdominal obesity includes subcutaneous and visceral adipose tissues, key for identifying IR. In this study, TyG, TyG-WC, and TyG-BMI proved to be efficient, suggesting that lipotoxicity and glucotoxicity are key in IR modulation ( 8 ).

We used the original formula Simental-Medía and cols. ( 6 ) proposed to identify the IR. It is important to disclose that this formula is still used even in 2021 and 2022 ( 53 - 58 ); however, regardless of the applied formula, the TyG has been described as an adequate surrogate marker for IR in children and adolescents around the world ( 58 - 64 ).

In summary, IR has been associated with obesity and other components of MS in adults as well as in children and adolescents. Therefore, TyG’s importance in discriminating IR stands out, which can be explained by the fact that one of the main mechanisms of IR modulation is glucolipotoxicity. TG, regardless of G, influences the results because hypertriglyceridemia is a cause and consequence of abnormal G metabolism ( 41 ). When ectopic lipid accumulates in the liver and skeletal muscle, the insulin binding receptor can prevent insulin action, leading to reduced hepatic glycogen synthesis and reduced uptake of muscle G. In other words, increased fatty acid oxidation limits the utilization of G by the action of insulin ( 65 ).

This study has some limitations. First, it was not possible to assess causality due to the study’s cross-sectional nature, and research that confirms the use of TyG in adolescents to predict the future occurrence of IR is necessary. Second, HOMA-IR is a validated and widely used method for the diagnosis of IR; however, it would be helpful if we could assess the indicators’ discriminatory power using a hyperinsulinemic-euglycemic clamp as a reference (gold standard test). Third, we used secondary data; however, we conducted rigorous quality assessment to minimize the possibility of bias. On the other hand, the appropriate sample yields high statistical power, on top of the fact that this was the first study that used TyG-related parameters (TyG-WC and TyG-BMI) in the adolescent population, which are this study’s main strengths.

In conclusion, TyG is a useful instrument for IR identification. This study suggests a cutoff point for the TyG index ≥ 7.94 for adolescents, ROC curve 0.64, which demonstrates moderate discriminative power. However, when added to anthropometric variables of excess weight (TyG-BMI) and visceral fat (TyG-WC), these indexes produced values above 0.79, increasing the potential use for diagnosis. The results point to TyG’s good discriminatory power for the diagnosis of IR in adolescents, especially when associated with BMI and WC.
